# Factors Interfering with Delineation on MRCP of Pancreaticobiliary Maljunction in Paediatric Patients

**DOI:** 10.1371/journal.pone.0154178

**Published:** 2016-04-22

**Authors:** Shun-gen Huang, Wan-liang Guo, Jian Wang, Mao Sheng, Xing-hao Lan, Lin Fang

**Affiliations:** 1 General surgery department, Children’s Hospital of Soochow University, Suzhou, China, 215003; 2 Radiology department, Children’s Hospital of Soochow University, Suzhou, China, 215003; Digestive Disease Research Center, Scott & White Healthcare, UNITED STATES

## Abstract

**Background:**

The aim of this study was to assess factors for delineating the pancreaticobiliary junction in the presence of pediatric congenital choledochal cysts (CCC) using Magnetic resonance cholangiopancreatography (MRCP).

**Methods:**

Retrospective review of medical records for 48 patients with CCC was conducted, including demographics, biliary amylase and MRCP findings if available. With univariate and multivariate logistic regression, we measured significant factors affecting pancreaticobiliary maljunction(PBM) diagnoses by MRCP.

**Results:**

Of the subjects enrolled with CCC. Twenty-eight cases had PBM according to MRCP. Univariate analysis confirmed that age, cyst diameter > 30 mm and cysts that descended to the introitus pelvis affected junctional delineation and detection of PBM (P<0.05). Stepwise logistic regression analysis confirmed large cysts in the introitus pelvis predicted pancreaticobiliary junctional delineation in MRCP and these data agreed with the literature. A correlation between cyst diameter and the length of the common channel was found as was cyst diameter and biliary amylase although there were no significant differences between them.

**Conclusions:**

Age, cyst diameter >30 mm and descending cysts into the introitus pelvis affected junctional delineation of the pancreatic and bile duct in PBM with MRCP. Large cyst descension into the introitus pelvis was an independent factors affecting PBM detection.

## Introduction

Pediatric pancreaticobiliary maljunction (PBM) is a rare congenital anomaly in which the main pancreatic and bile ducts are joined outside the duodenal wall and form a long common channel. The sphincter of Oddi in PBM cannot control bile and pancreatic juice flow, and this can lead to two-way reflux of bile and pancreatic juice [1,2] which can cause cholestasis, stone formation, pancreatitis, hyperplasia and epithelial atypical growth of the bile duct or gallbladder, as well as tumor formation [3,4,5,6]. Many PBM cases are accompanied with bile duct dilation, and identifying the precise anatomy of the pancreaticobiliary junction would help differentiate PBM from congenital choledochal cysts (CCC) and biliary atresia with cystic dilation [7].

Imaging can help delineate the junction of the common bile and pancreatic ducts and can diagnose PBM. Endoscopic retrograde cholangiopancreatography (ERCP) can also delineate the pancreaticobiliary junction [8, 9], but it is invasive and can cause pancreatitis. Magnetic resonance cholangiopancreatography (MRCP), in contrast, is non-invasive and efficient for evaluating pediatric pancreaticobiliary junctions [10, 11]. Because the pediatric pancreatic duct is slim, delineation is difficult and PBM diagnoses are challenging [7,12].

Few studies have focused on factors affecting pancreaticobiliary junction delineation by MRCP. So, from January 2009 until December 2014 we retrospectively studied clinical and MRCP features of cases and measured bile duct amylase for pediatric subjects with common bile duct dilation, focusing on factors that would modify diagnosis of pediatric PBM in MRCP.

## Materials and Methods

### Study Subjects

The study protocol was approved by the Institutional Ethics Review Committee at Children’s Hospital of Soochow University. Informed consent was signed by subject guardians. All experiments were carried out in strict accordance with the institution guidelines regarding the acquisition and experimental use of human tissues. We retrospectively reviewed the medical records of 48 patients common bile duct dilation hospitalized between January 2009 and December 2014. Inclusion criteria of PBM was 1) the common channel is longer than 5 mm by MRCP and combined by intraoperative cholangiography(IOC); 2) patients with 5 mm or longer common channel had biliary amylase(obtained by IOC) >1,000 U/L. If the cases do not meet these criteria, then the cases were diagnosed as CCC.

### MRCP

MRCP was performed before diagnosing of PBM in all cases. Before MRCP, all subjects were maintained in jejunitas for 4 h then MRCP was performed with sedation for subjects 10 years-of-age or younger. A Symphony 1.5 T scanner (Siemens, Erlangen, Germany) with an abdominal phased array coil was used as follows: T1-weighted and T1-weighted fast spin series (field of view 24–28 cm, repetition time [TR] 173 ms, echo time [TE] 2.64 ms, flip angle 70, matrix 256 * 128, radiofrequency (RF) bandwidth 260 Hz/Px) and a T2-weighted sequence (TR 1,000 ms, TE 60 ms, RF bandwidth 230 Hz/Px). For MRCP, half-Fourier acquisition single shot turbo spin echo (HASTE) was used with multilayer thin coronal and axial T2-weighted imaging (TR 1,200 ms, TE 80 ms, slice thickness 4 mm). Oblique thick slabs were acquired in the planes of the common bile and pancreatic ducts. For multi-angle imaging, TR was 4,500 ms, TE 950 ms and slice thickness 60 mm were used.

Two radiologists who were unaware of the pathological findings independently reviewed the images and reached consensus through discussion. A diagnosis of PBM was established if the common channel is longer than 5 mm. They also assess the shape of the intrahepatic bile duct and gallbladder, pancreatitis, surgical pathology, symptom profiles, operative notes and pathological records were compared with the imaging findings.

### Factor Analysis

Forty-eight CCC patients were assessed for gender, age, biliary amylase and MRCP findings and two groups were established: PBM and non-PBM. Within these groups, age, gender, common bile duct shape, distal position of papilla of Vater, cyst stones and cyst size, gallbladder and pancreatic duct dilation and CCC were assessed as was stomach, duodenal, and small bowel fluid. MRCP helped classify cysts as being smaller or larger than 30 mm. Ages were grouped as infant (younger than one year-of-age) or pediatric (older than one year-of-age) as well. Stomach fluid was documented to occur in the gastric fundus or body and duodenal fluid was defined as occurring in the second, third, or fourth duodenal portion and small bowel fluid was defined as occurring in the lower left or the lower and middle abdominal area. Pancreatic duct dilation was defined as dilation greater than 1 mm. Descending cysts were obviously apparent in the introitus pelvis.

### Statistical Analysis

Data are presented as number (*n*) and percentage. Univariate comparisons were made using a nonparametric one-way Wilcoxon rank sum, a χ^2^, or Fisher’s exact test; depending on statistical distribution. To evaluate risk factors affecting diagnosing PBM by MRCP, a stepwise logistic regression analysis was performed with SAS 8. 0. P<0.05 were considered statistically significant.

## Results

**Fig 1:**
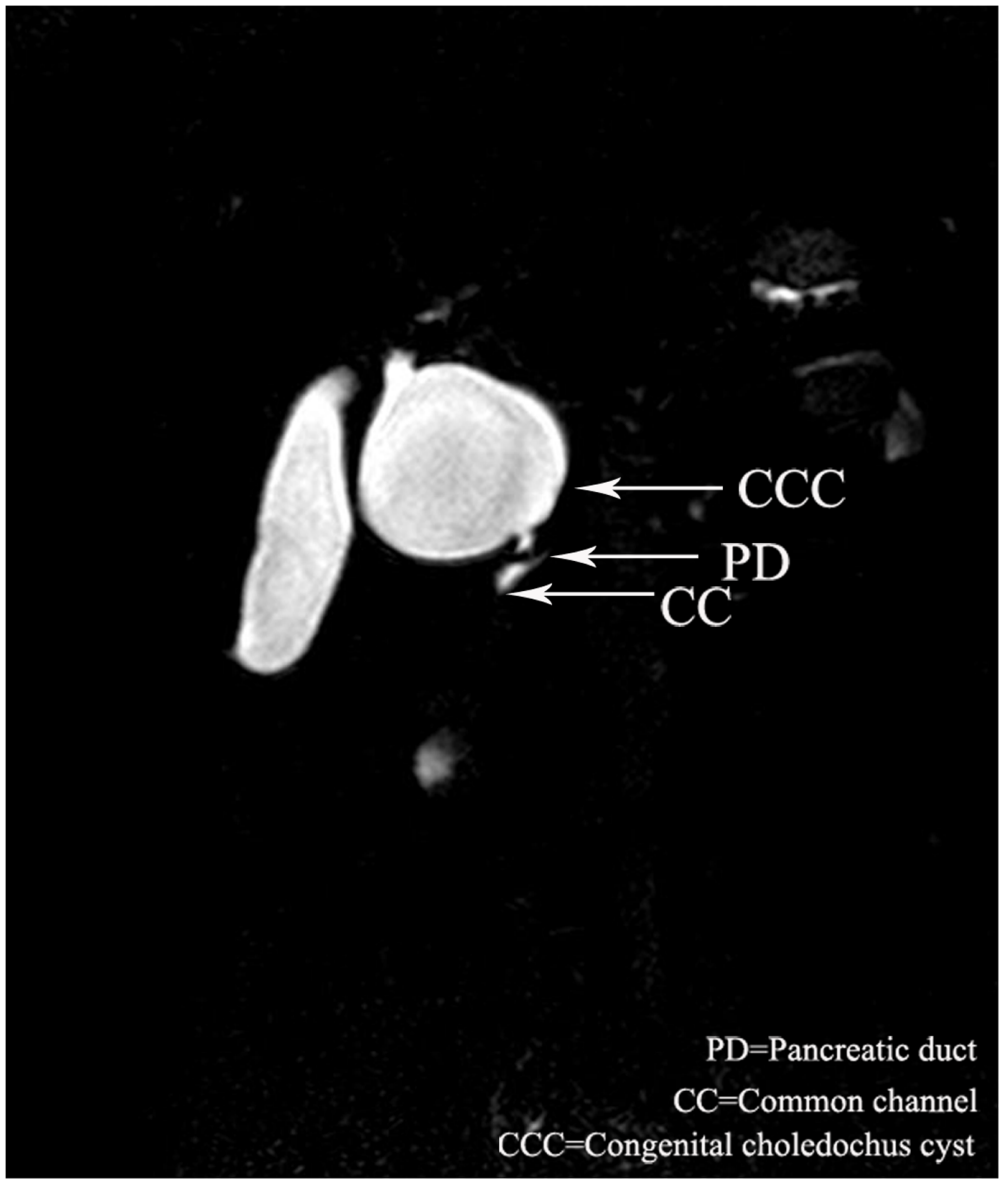
A 2-year old female with PBM, MRCP showed the spheroidal dilataion of the cyst.

**Fig 2:**
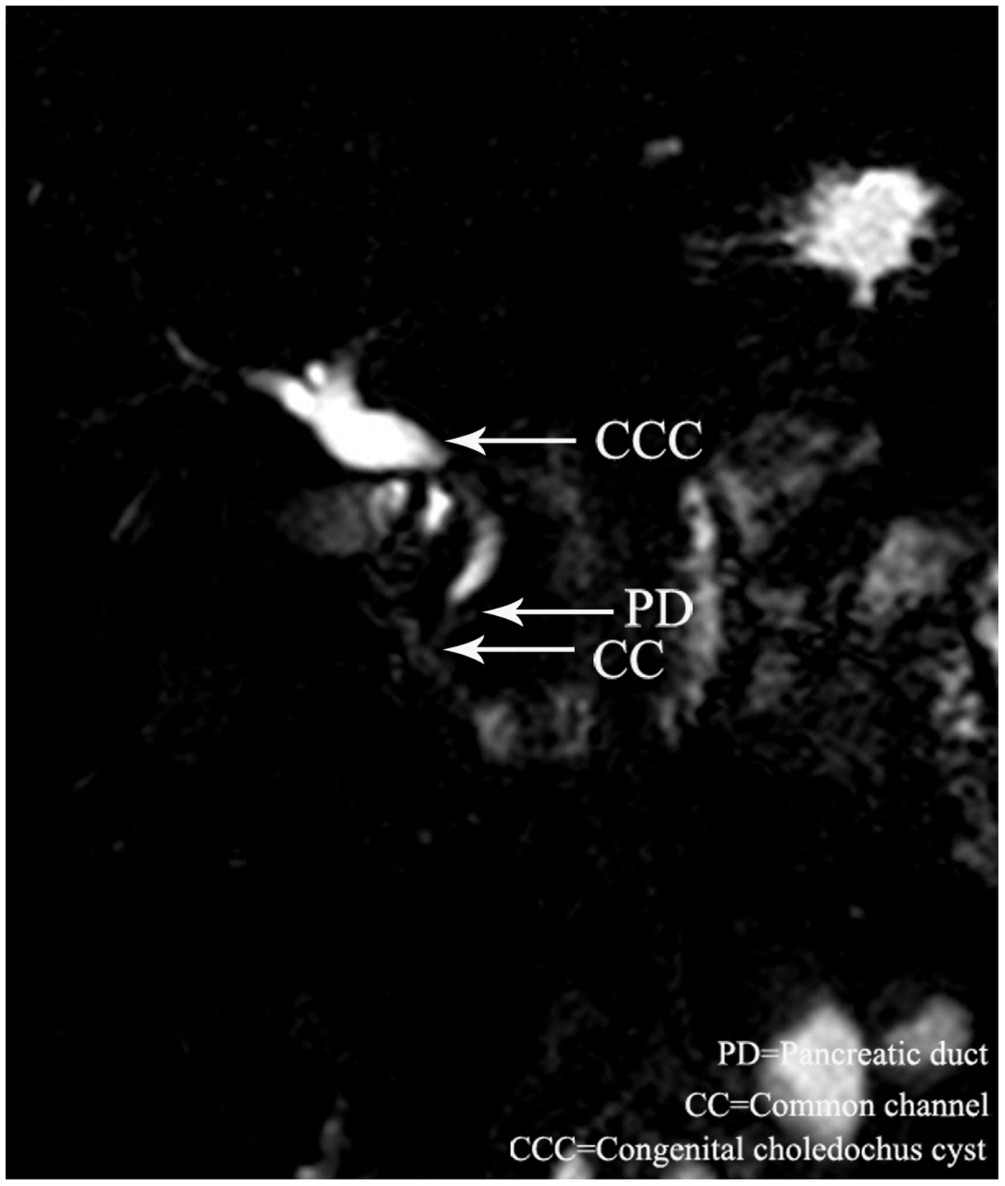
A 2-year old male with PBM, MRCP showed the fusiformis dilataion of the cyst.

**Fig 3:**
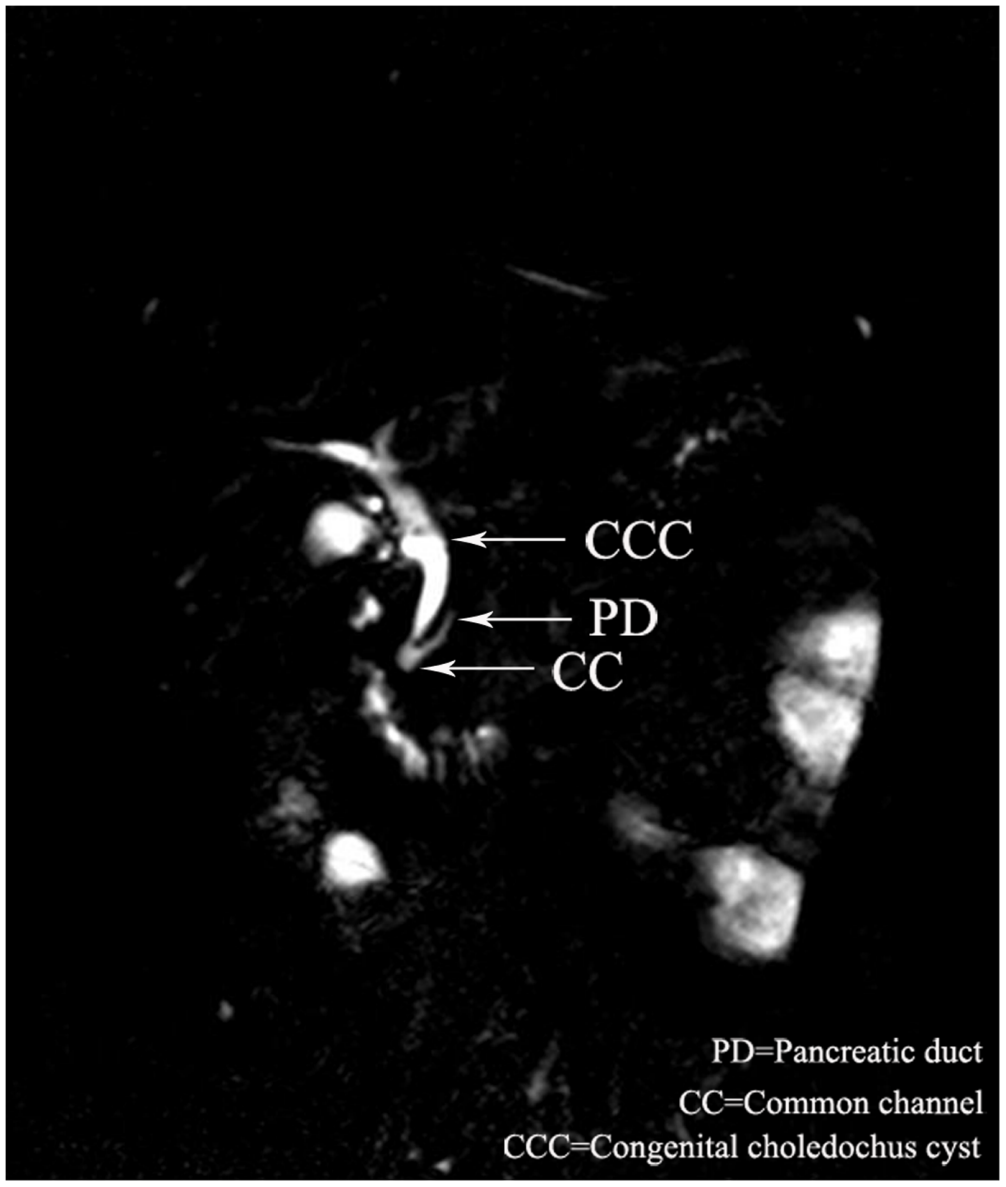
A 9-year old female with PBM, MRCP showed the cylindricality dilataion of the cyst.

**Fig 4:**
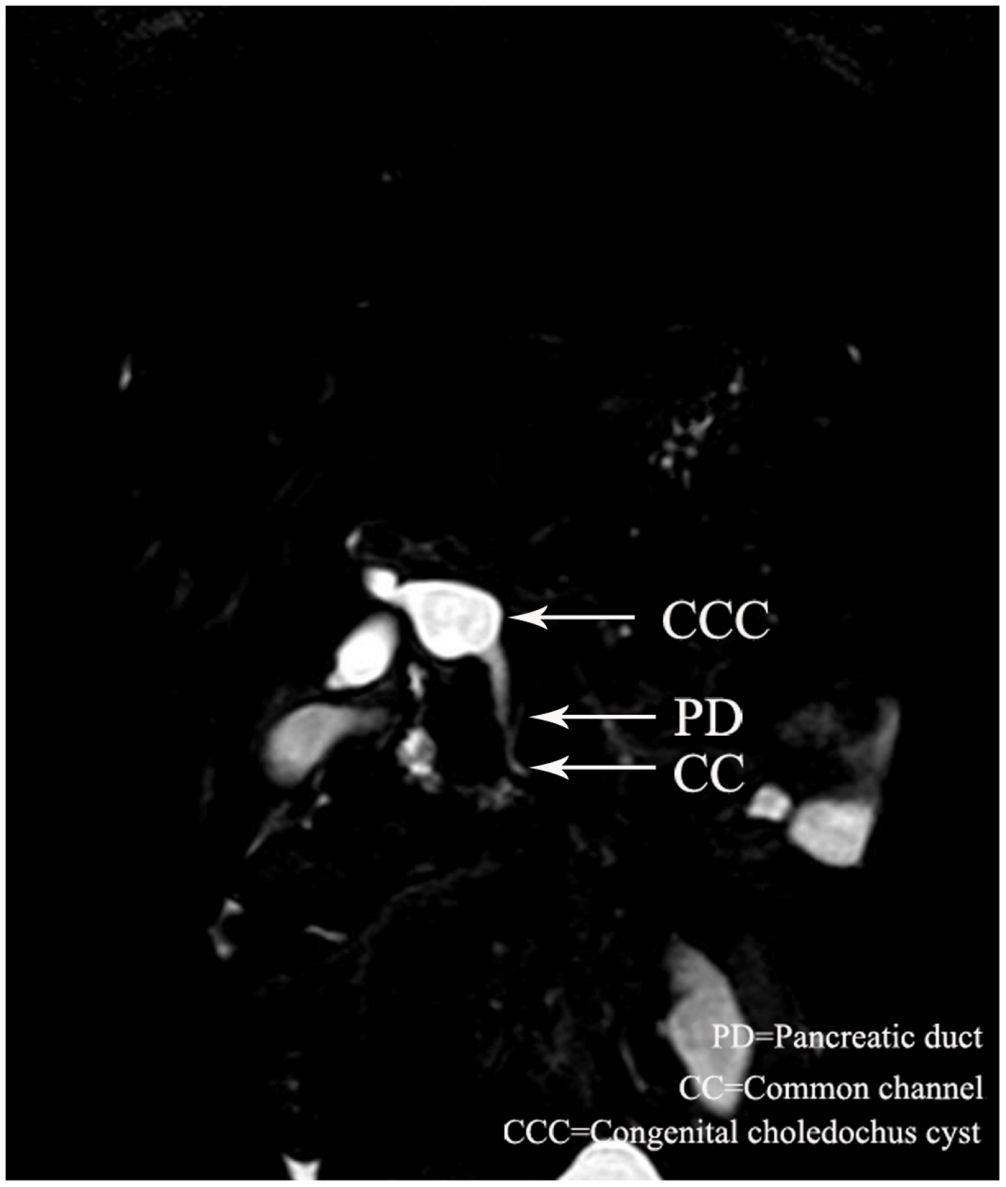
A 3-year old female with PBM, MRCP showed the gourd dilataion of the cyst.

**Fig 5:**
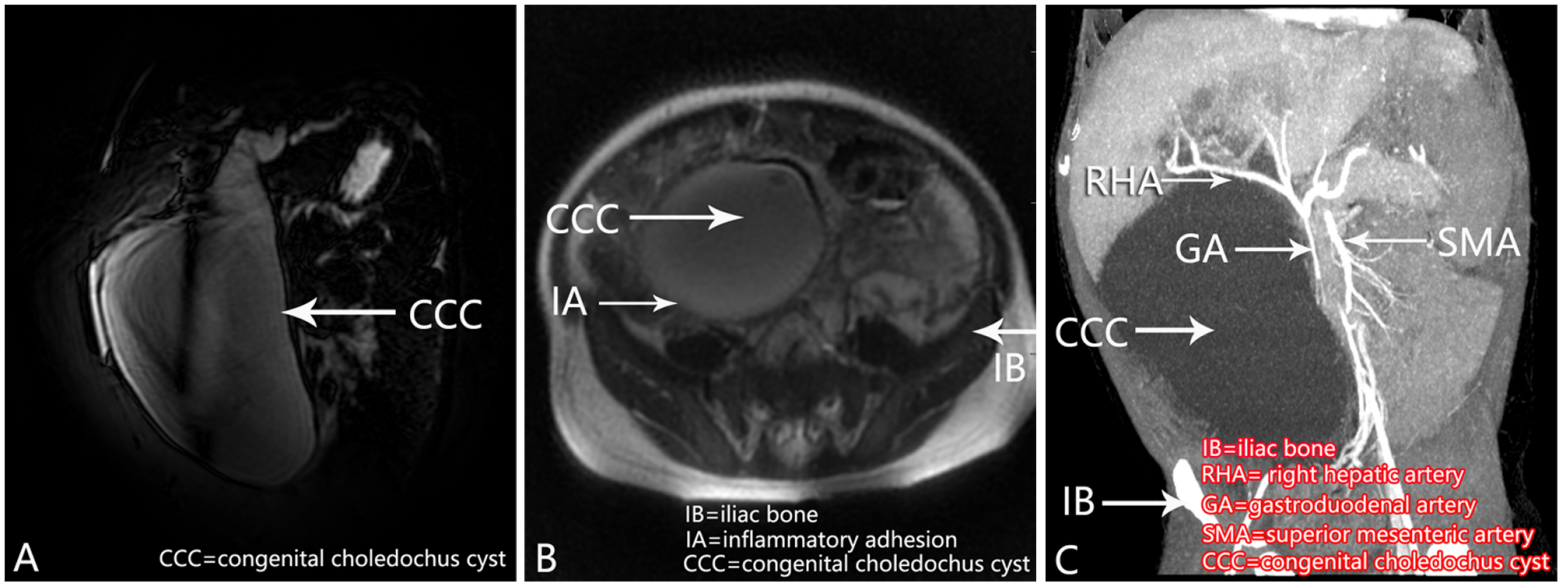
A 1-year old female with cyst descension into the introitus pelvis. A, Pancreatic and common channel was not detected in MRCP. B, Axis T2WI imaging showed the cyst descension into introitus pelvis. Inflammatory adhesion can be found in the lower-right side of the cyst. C, contrast-enhanced multislice spiral computed tomography (multiplanar reformation imaging) showed the cyst occupies large intra-abdominal space, displacement of vessels, modified the pelvic and duodenal anatomy.

Overall, there were 18(37.5%) male and 30 (62.5%) female patients with dilatation of bile duct performed MRCP. The median age of the 48 patients was 3 years (range, 6 days to 13 years). Twenty-eight cases were defined as PBM by MRCP. The common channel raging from 5.5 to 25 mm(avage,13.0mm). The biliary amylase concentration ranging from 1306 to 62978U/L(avage,28206 U/L). The shape of cyst included spheroidal (Fig 1) in 20 cases, fusiformis(Fig 2) in 6 cases, cylindricality(Fig 3) in 12 cases, gourd(Fig 4) in 2 cases and cyst descension into the introitus pelvis(Fig 5) in 8 cases. Thirty-eight cases showed the distal position of papilla of Vater in the third portion of the duodenum.

Table 1 depicts MRCP and clinical characteristics of the patients. Univariate analysis revealed that infancy, cysts > 30 mm and cyst descension into the introitus pelvis affected pancreaticobiliary junction delineation and detection of PBM (P<0.05). No other factors affected diagnoses. (*P*>0.05; see Table 1). A stepwise logistic regression analysis of 48 cases was performed to assess independent predictors affecting pancreaticobiliary junction delineation in MRCP. Again, cyst descension into the introitus pelvis was the most important (see Table 2). These data agreed with Hosmer and Lemeshow’s goodness-of-fit test (P = 0.6182). Correlation analyses of cyst diameter and common channel length as well as cyst size and biliary amylase concentration were not significant (P>0.05) (Table 3).

**Table 1:** Clinical and MRCP characteristics in relation to diagnosis PBM.

varable	PBM	Non-PBM	P value
	(28 cases)	(20 cases)	
The shape of the cyst			
Spheroidal	13(46.4%)	7(35.0%)	0.4285
Fusiformis	6(21.4%)	1(5.0%)	0.2138
Cylindricality	7(25.0%)	4(20.0%)	0.7538
Gourd	2(7.1%)	-(0%)	0.5035
Cyst descension into the introitus pelvis	-(0%)	8(40.0%)	0.0032
Diameter of the cyst >30mm	8(28.6%)	13(65.0%)	0.0121
Stone in the cyst	8(28`6%)	3(15.0%)	0.3190
Dilatation of gallbladder	15(53.6%)	10(50.0%)	0.8071
CCC type (type I)	16(57.1%)	8(40.0%)	0.2416
Fluid in gastric fundus	25(89.3%)	13(65.0%)	0.0700
Fluid in the second portion of duodenum	25(89.3%)	13(65.0%)	0.0700
Fluid in the left lower part of small bowel	18(64.3%)	12(60.0%)	0.7624
Dialation of main pancreatic duct	3(10.7%)	- (0%)	0.2553
Gender(F)	17(60.7%)	13(65.0%)	0.7624
Infant	2(7.1%)	8(40.0%)	0.0100

**Table 2:** Stepwise logistic regression model for significant affecting factors of detection of PBM by MRCP.

variable	Chi-Square	OR	95%Wald Conficence Limits	P value
Cyst descension into the introitus pelvis	5.2725	0.135	0.024–0.746	0.0217
Diameter of the cyst >30 mm	0.0583	0.825	0.174–3.916	0.8082
Infant	0.3305	0.586	0.095–3.629	0.5654

Hosmer and Lemeshow Goodness-of-Fit Test (p = 0.6182)

**Table 3:** Correlation between diameter of cyst and related factors.

variable	Spearman correlation coefficients	
	r_s_	P value
Biliary amylase concentration	0.30285	0.1502
The length of common channel	0.64286	0.1194

## Discussion

### General finding in MRCP

In clinical practice, radiographic detection of common channel length is key to diagnosing PBM but this is difficult. Routing CT cannot directly delineate the long common channel and ERCP; although invasive, it can confirm the common channel. Miyazaki’s group reported that MRCP with a half-Fourier acquisition single-shot spin-echo (HASTE) sequence is noninvasive and can evaluate biliary or pancreatic disease in young children [10,13,14]. However, detecting PBM with MRCP is challenging in young patients and cases of dilated common bile ducts. Reports suggest diagnosis of pediatric PBM ranges from 40–80% with MRCP [15,16]. Thus, we used HASTE to confirm 66.7% of PBM diagnosis by MRCP. In fourteen PBM cases, MRCP could not confirm the long common channel, a finding that agrees with previous studies [10,14]. We evaluated 38 PBM cases with distal position of papilla of Vater in the distal duodenum, and these cases were similar to those of Li [17]. PBM cases often associate with distal position of papilla of Vater in distal duodenal portion.

### Related factors for detecting PBM in MRCP

To investigate potential factors that may affect identifying pancreatic ducts, we described MRCP and features for detecting pediatric PBM. Univariate analysis indicated that patient age, cyst size and cyst descent into the introitus pelvis may affect pancreaticobiliary junction delineation and PBM diagnoses. Suzuki’s group reported that cyst dilation and patient age affected pancreaticobiliary junction identification and PBM confirmation [18]. We found no significant difference among MRCP findings as detailed in the Results. Multiple logistic regression confirmed that only large cysts that descended into the introitus pelvis were independent factors for pancreaticobiliary junction identification and PBM diagnosis for pediatric cases.

In the present study, 8 cases with large and descended cysts had undetectable pancreatic ducts and common channels, and the duodenum could not be delineated. Thus, large and descended cysts occupies large intra-abdominal space and have modified the pelvic and duodenal anatomy. Displacement of vessels, severe inflammatory adhesion and severe biliary obstruction may be the reasons for the descended cysts[19]. And this made the pancreatic ducts and common channels can not be detected. Saito’s group [20] reported that for 16 pediatric CCCs, PBM was identified 56% by MRCP. Yamataka and colleagues [21] reported that cyst size and location prevented visualization of PBM with CCCs and Kamisawa and colleagues [22] agreed. Also, the sphincter of Oddi’s spasm can cause an artificially short common channel length [23,24]. For the 3 cases for which the common channel could not be identified and without biliary amylase level, CCC may be the cause.

For 8 of our infant patients, we could not detect the main pancreatic duct with MRCP due to small size (<1 mm). Siles’ group [25] reported 16 MRCP pediatric cases of children younger than 3 months-of-age and they also identified main pancreatic ducts in 2 cases. Fitoz’s group [26] reported that young age can hinder pancreaticobiliary maljunction identification.

Cyst diameter can complicate pancreatic duct identification by MRCP. With cysts larger than >30 mm detection decreased and distal common bile duct areas were not identified in 7 cases with MRCP. Also, dilated bile ducts exceeding 30 mm complicated identification by MRCP because the overlapping bottom edge of the large CCC obscured views [18,21]. Sugiyama’s group reported [27] similar data for pediatric and adult patients for which large and descended cysts were most prominent. Gwal’s group [12] reported cases of pancreatic and biliary disease in which main pancreatic ducts were identified in 80.5% cases suggesting that common bile duct dilation affects main pancreatic duct identification. We found no correlation between cyst size and common channel length or bile amylase, suggesting that cysts can occlude the distal areas of the common bile duct.

### Limitations

This study is limited by being a small-sample retrospective study and selection bias may be a factor. Also, only HASTE MRCP was used and no controls were involved. A prospective study with more subjects is needed to further evaluate MRCP in pediatric cases.

## Conclusions

In conclusion, 66.7% of pediatric PBM cases with CCC can be diagnosed by HASTE MRCP, and there was no correlation between cyst dilatation and common channel length or biliary amylase. Patient age, cyst diameters>30 mm and cyst descension into the introitus pelvis affected junctional delineation and detection of PBM. Large cyst descension into the introitus pelvis was an independent factors affecting pancreatic and bile duct junctional delineation and PBM detection with this technique.

## Supporting Information

S1 Table Patient baseline and MRCP characteristics in relation to diagnosis PBM. (DOC)
