# Supplementary material for: Factors Interfering with Delineation on MRCP of Pancreaticobiliary Maljunction in Paediatric Patients
Source: PLoS One. 2016 Apr 22;11(4):e0154178. doi: 10.1371/journal.pone.0154178 (PMC4841599; doi:10.1371/journal.pone.0154178)
Supplement: S1 Table — (DOC) [file pone.0154178.s001.doc]

Patient baseline and MRCP characteristics in relation to diagnosis PBM

| Study Subjects | gender | age | PBM | The shape of the cyst | Diameter of the cyst (mm) | Stone in the cyst | Dilatation of gallbladder | CCC type | Fluid in gastric fundus | Fluid in the second portion of duodenum | Fluid in the left lower part of small bowel | Dialation of main pancreatic duct |
| --- | --- | --- | --- | --- | --- | --- | --- | --- | --- | --- | --- | --- |
| 1 | F | 2Y | 1 | Spheroidal | 39 | 0 | 1 | Ⅰ | 0 | 0 | 0 | 0 |
| 2 | F | 1Y | 1 | Spheroidal | 27 | 0 | 0 | Ⅳ | 1 | 1 | 1 | 0 |
| 3 | F | 2Y | 1 | Spheroidal | 18 | 1 | 1 | Ⅳ | 1 | 1 | 1 | 0 |
| 4 | F | 5Y | 1 | Spheroidal | 28 | 0 | 0 | Ⅳ | 1 | 1 | 1 | 0 |
| 5 | M | 3Y | 1 | Spheroidal | 50 | 0 | 0 | Ⅰ | 0 | 0 | 0 | 0 |
| 6 | F | 6Y | 1 | Spheroidal | 50 | 1 | 1 | Ⅳ | 1 | 1 | 1 | 0 |
| 7 | M | 2Y | 1 | Spheroidal | 45 | 0 | 1 | Ⅰ | 1 | 1 | 0 | 1 |
| 8 | M | 10Y | 1 | Spheroidal | 52 | 1 | 0 | Ⅳ | 1 | 1 | 1 | 1 |
| 9 | F | 5Y | 1 | Spheroidal | 20 | 0 | 0 | Ⅰ | 1 | 1 | 0 | 0 |
| 10 | M | 2Y | 1 | Spheroidal | 35 | 0 | 0 | Ⅰ | 1 | 1 | 1 | 0 |
| 11 | F | 4Y | 1 | Spheroidal | 20 | 1 | 1 | Ⅰ | 1 | 1 | 0 | 0 |
| 12 | F | 3Y | 1 | Spheroidal | 25 | 0 | 0 | Ⅰ | 1 | 1 | 0 | 0 |
| 13 | M | 2Y | 1 | Spheroidal | 31 | 0 | 0 | Ⅰ | 1 | 1 | 0 | 0 |
| 14 | M | 3Y | 1 | Cylindricality | 5 | 0 | 1 | Ⅰ | 1 | 1 | 1 | 0 |
| 15 | F | 2Y | 1 | Cylindricality | 11 | 1 | 0 | Ⅰ | 1 | 1 | 1 | 0 |
| 16 | M | 5Y | 1 | Cylindricality | 4 | 0 | 1 | Ⅰ | 1 | 1 | 1 | 0 |
| 17 | M | 13Y | 1 | Cylindricality | 22 | 0 | 1 | Ⅳ | 1 | 1 | 0 | 0 |
| 18 | F | 9Y | 1 | Cylindricality | 20 | 0 | 1 | Ⅳ | 1 | 1 | 1 | 0 |
| 19 | F | 1Y | 1 | Cylindricality | 5 | 1 | 1 | Ⅰ | 1 | 1 | 1 | 0 |
| 20 | M | 3Y | 1 | Cylindricality | 11 | 0 | 1 | Ⅳ | 1 | 1 | 1 | 0 |
| 21 | F | 2Y | 1 | Fusiformis | 13 | 0 | 1 | Ⅳ | 1 | 1 | 1 | 0 |
| 22 | M | 2Y | 1 | Fusiformis | 9 | 0 | 1 | Ⅰ | 1 | 1 | 1 | 0 |
| 23 | F | 6Y | 1 | Fusiformis | 15 | 0 | 0 | Ⅰ | 1 | 1 | 1 | 0 |
| 24 | F | 9Y | 1 | Fusiformis | 14 | 0 | 0 | Ⅰ | 1 | 1 | 1 | 1 |
| 25 | F | 4Y | 1 | Fusiformis | 15 | 1 | 1 | Ⅰ | 0 | 0 | 0 | 0 |
| 26 | M | 3Y | 1 | Fusiformis | 8 | 0 | 1 | Ⅳ | 1 | 1 | 1 | 0 |
| 27 | F | 5Y | 1 | Gourd | 31 | 0 | 0 | Ⅳ | 1 | 1 | 1 | 0 |
| 28 | F | 3Y | 1 | Gourd | 17 | 1 | 0 | Ⅳ | 1 | 1 | 0 | 0 |
| 29 | F | 8Y | 0 | Spheroidal | 53 | 0 | 0 | Ⅳ | 0 | 0 | 1 | 0 |
| 30 | F | 3Y | 0 | Spheroidal | 28 | 0 | 1 | Ⅳ | 1 | 1 | 1 | 0 |
| 31 | F | 11Y | 0 | Spheroidal | 68 | 0 | 0 | Ⅰ | 1 | 1 | 1 | 0 |
| 32 | F | 3Y | 0 | Spheroidal | 29 | 0 | 1 | Ⅰ | 0 | 0 | 0 | 0 |
| 33 | M | 6Y | 0 | Spheroidal | 35 | 1 | 1 | Ⅰ | 0 | 0 | 0 | 0 |
| 34 | F | 6Y | 0 | Spheroidal | 40 | 0 | 0 | Ⅰ | 1 | 1 | 1 | 0 |
| 35 | M | 3M | 0 | Spheroidal | 35 | 1 | 1 | Ⅰ | 1 | 1 | 1 | 0 |
| 36 | F | 2Y | 0 | Cylindricality | 16 | 0 | 1 | Ⅰ | 1 | 1 | 0 | 0 |
| 37 | F | 4Y | 0 | Cylindricality | 17 | 1 | 1 | Ⅳ | 1 | 1 | 1 | 0 |
| 38 | M | 1Y | 0 | Cylindricality | 6 | 0 | 1 | Ⅳ | 1 | 1 | 1 | 0 |
| 39 | F | 5Y | 0 | Cylindricality | 10 | 0 | 1 | Ⅳ | 1 | 1 | 1 | 0 |
| 40 | F | 2Y | 0 | Fusiformis | 11 | 0 | 1 | Ⅰ | 1 | 1 | 1 | 0 |
| 41 | F | 2Y | 0 | Cyst descension into the introitus pelvis | 73 | 0 | 0 | Ⅳ | 0 | 0 | 0 | 0 |
| 42 | F | 1Y | 0 | Cyst descension into the introitus pelvis | 42 | 0 | 0 | Ⅳ | 1 | 1 | 0 | 0 |
| 43 | M | 5Y | 0 | Cyst descension into the introitus pelvis | 68 | 0 | 0 | Ⅳ | 1 | 1 | 0 | 0 |
| 44 | M | 2M | 0 | Cyst descension into the introitus pelvis | 37 | 0 | 0 | Ⅳ | 1 | 1 | 0 | 0 |
| 45 | M | 6D | 0 | Cyst descension into the introitus pelvisl | 65 | 0 | 0 | Ⅰ | 0 | 0 | 0 | 0 |
| 46 | M | 1Y | 0 | Cyst descension into the introitus pelvis | 71 | 0 | 0 | Ⅳ | 0 | 0 | 1 | 0 |
| 47 | F | 1Y | 0 | Cyst descension into the introitus pelvis | 80 | 0 | 1 | Ⅳ | 1 | 1 | 1 | 0 |
| 48 | F | 10D | 0 | Cyst descension into the introitus pelvis | 31 | 0 | 0 | Ⅳ | 0 | 0 | 1 | 0 |

* 1=positive, 0=negative,

* gender( F=female, M=male),

* age( D=day, M=month, Y=year)
